# Oral Administration of Liposomal Resveratrol for Wound Healing in a Zebrafish Model

**DOI:** 10.3390/molecules31091379

**Published:** 2026-04-22

**Authors:** Ruei-Siang Yu, Minh-Quan Tran, Mei-Wen Tseng, Chung-Der Hsiao, Hung-Maan Lee, Ming-Fa Hsieh

**Affiliations:** 1 Department of Biomedical Engineering, Chung Yuan Christian University, No. 200, Zhongbei Rd., Zhongli Dist., Taoyuan 320314, Taiwanminhquan0141@gmail.com (M.-Q.T.);; 2 Department of Pharmacy, Hualien Armed Forces General Hospital, No.163, Jiali Rd., Xincheng Township, Hualien 971051, Taiwan; 3 School of Pharmacy, College of Pharmacy, National Defense Medical University, No. 161, Sec. 6, Minquan Rd., Neihu Dist., Taipei 11490, Taiwan; 4 Department of Biosciences, Chung Yuan Christian University, No. 200, Zhongbei Rd., Zhongli Dist., Taoyuan 320314, Taiwan; cdhsiao@cycu.edu.tw; 5 School of Medicine, Tzu Chi University, No. 701, Sec. 3, Zhongyang Rd., Hualien 970004, Taiwan

**Keywords:** resveratrol, liposomes, zebrafish, wound healing, oral delivery

## Abstract

Wound healing research has advanced through nanotechnology-based delivery systems that enhance the stability and therapeutic potential of bioactive compounds. Resveratrol, a natural polyphenol with antioxidant and anti-inflammatory properties, shows promise for wound healing but is limited by poor bioavailability. This study investigates the efficacy of nano-liposome-encapsulated resveratrol in enhancing skin wound repair in adult zebrafish (*Danio rerio*). Using a laser-based ablation method, precise full-thickness skin wounds were induced and monitored over 50 days. Resveratrol-loaded liposomes were prepared and orally administered via gavage to facilitate systemic exposure. Compared to the control and blank liposome groups, resveratrol liposome treatment significantly accelerated wound closure, achieving earlier healing milestones (25%, 50%, and 75%). The zebrafish model provided a regenerative platform for real-time evaluation of nanomedicine-based therapies. This study demonstrates the wound healing effects of resveratrol and liposomal encapsulation, offering a targeted, systemically administered strategy for advanced systemic healing and highlighting zebrafish as a valuable model for preclinical regenerative medicine research.

## 1. Introduction

Liposome technology has emerged as a key advancement in drug delivery, offering a versatile platform for encapsulating and delivering therapeutic agents. Since their development in the 1970s, liposomes, which are spherical vesicles composed of one or more phospholipid bilayers, have been widely applied in advanced drug delivery systems, including wound healing therapies. Their amphiphilic nature enables the encapsulation of both hydrophilic and lipophilic compounds, thereby improving drug solubility, stability, and bioavailability [1,2]. This structural design supports controlled and targeted drug release, minimizing systemic toxicity and enhancing therapeutic efficacy. Liposomes’ membrane-like characteristics also facilitate interactions with biological systems, leading to improved tissue penetration and sustained drug release [1]. Over time, various liposomal types have been developed, such as long-circulating, stealth, and stimuli-responsive liposomes, each engineered to optimize delivery and reduce side effects [3]. Advances in bioconjugation have further enabled targeted delivery to specific cells or tissues, which is especially valuable in wound healing.

Resveratrol (RSV), a polyphenolic compound found in red wine, grapes, and berries, has gained attention for its role in promoting wound healing. Its potent antioxidant and anti-inflammatory properties help reduce oxidative stress and inflammation, which are key barriers to tissue repair in conditions such as diabetic ulcers and surgical wounds [4,5,6]. RSV enhances angiogenesis, stimulates fibroblast proliferation, and modulates growth factor expression, all of which are critical for regeneration [7,8].

Despite RSV’s regenerative potential, its low bioavailability limits its effectiveness; almost all RSV was metabolized after oral administration in human subjects [9]. To overcome this, nanoparticle-based delivery systems have been developed. For example, RSV-loaded nanoparticles significantly enhance oral bioavailability, enabling detectable plasma levels of RSV and its active metabolites [10]. These delivery strategies not only improve RSV’s bioavailability but also expand its clinical potential. Additionally, formulations such as fibrous scaffold and RSV conjugates have been explored to maximize wound-healing potential [11,12]. Furthermore, studies indicate that RSV extends beyond antioxidant effects, as it also influences key signaling pathways involved in inflammation and cell migration, which are essential for regulating cellular responses to stress and promoting tissue repair [8,13].

Zebrafish (*Danio rerio*) have emerged as a pivotal model in regenerative medicine due to their remarkable ability to regenerate tissue without scarring, unlike mammals [14,15]. Although zebrafish share the major wound healing phases of hemostasis, inflammation, re-epithelialization, granulation, and remodeling with mammals, differences in skin architecture, such as the absence of hair follicles and a multilayered dermis, and variations in immune system complexity may influence therapeutic responses, highlighting that findings in zebrafish require cautious interpretation when translating to mammals. Despite these differences, zebrafish still can serve as an effective high-throughput platform for screening wound-healing agents, including nanoparticles, plant extracts, and biomaterials [15,16]. Laser-based skin ablation enables the creation of precise, minimally invasive wounds, improving reproducibility compared with traditional injury methods [17,18]. Zebrafish are well-suited for oral drug administration that mimics human intake, with gavage techniques enhancing dose control and reducing stress [19]. The strong genetic conservation of wound-healing pathways between zebrafish and humans supports their translational relevance for developing novel therapeutic interventions [15,20], collectively positioning zebrafish as a powerful model for advancing wound repair research and drug discovery.

This study investigates the use of liposome-encapsulated RSV in a zebrafish wound model to enhance RSV stability, bioavailability, and controlled delivery. By leveraging the regenerative capacity of zebrafish, laser-based wound induction, and oral administration, the therapeutic potential of RSV liposomes for promoting effective wound healing was evaluated.

## 2. Materials and Methods

### 2.1. Fabrication of Liposomes

The fabrication method used in this study has been published elsewhere [21]. Briefly, liposomal formulations containing resveratrol (RSV, Tokyo Chemical Industry, Col, Ltd., Tokyo, Japan) were prepared using the thin-film hydration method, followed by extrusion. The liposomes utilized either neutral L-α-phosphatidylcholine (PC, Sigma-Aldrich, St. Louis, MO, USA) or negatively charged 1,2-dioleoyl-sn-glycero-3-phospho-L-serine (PS, NOF Corporation, Tokyo, Japan) as the primary lipid components, in combination with cholesterol (CH, Sigma-Aldrich, St. Louis, MO, USA). Stock solutions of lipids and RSV at 10 mg/mL were prepared separately. For the final liposome formulations listed in Table 1, aliquots of the stock solutions were uniformly mixed in ethanol. The organic solvent was then removed using a rotavapor (N-1000, Eyela, Tokyo, Japan), resulting in a thin film on the wall of the round-bottom flask. The thin film was hydrated with deionized water, followed by ultrasonic oscillation at 60 °C for 1 h to produce a liposome solution at 1 mg/mL. The solutions were subsequently processed using an extruder (Avanti Polar Lipids, Alabaster, AL, USA) at 60 °C, subjected to seven repeated cycles of extrusion through polycarbonate filter membranes (Avanti Polar Lipids) with pore sizes of 400 nm and 200 nm to achieve a uniform particle size. The final formulations were purified using dialysis tubing (Spectrum™ Spectra/Por™ 3 RC dialysis membrane tubing MWCO = 3500 Da, New Brunswick, NJ, USA) against deionized water to remove unencapsulated reagents.

### 2.2. Characterizations of Liposomes

Three liposomal formulations were assessed for particle size and drug loading capacity (PC-RSV liposome). PC and PS liposomes were blank liposomes, while PC-RSV liposomes consisted of PC liposomes loaded with RSV.

#### 2.2.1. Particle Size, Size Distribution, and Zeta Potential Analysis

Dynamic light scattering (DLS) was employed to determine the average particle size, size distribution, and surface charge of the liposomes. A Zetasizer Ultra (Malvern, UK) was used to measure particle size and the polydispersity index (PI). The instrument analyzes the movement of liposomes undergoing Brownian motion using a source laser with a wavelength of 633 nm at a temperature of 25 °C. A detector positioned at a side angle (90° with respect to the source laser) was used to collect the scattered signals. Freshly prepared liposomes (0.1 mL) were diluted with 0.9 mL of deionized water. Each measurement was repeated three times. The instrument was calibrated with NanosphereTM Size Standards (Nominal diameter 20 nm, Cat. No: 3020A, Thermo Scientific, Santa Clara, CA, USA). For surface charge (Zeta Potential) measurements of the liposomes, the Zetasizer Ultra was employed, with samples loaded into capillary cuvettes (DTS1080, Malvern Panalytical Ltd., Malvern, UK).

#### 2.2.2. RSV Encapsulation Efficiency and Loading Capacity

The liposome’s capacity to encapsulate RSV was quantified using drug load efficiency (DLE) and drug load content (DLC). To determine the total amount of encapsulated RSV, 0.1 mL of PC-RSV liposome was mixed with 0.9 mL of 95 wt% ethanol, which disrupted the liposome and released the loaded RSV. The amount of RSV was subsequently measured using a UV/Vis spectrophotometer (UV-1800, Shimadzu, Kyoto, Japan). The measured absorbance value was used to calculate RSV concentration by comparing it with an established RSV standard curve obtained at the characteristic absorption peak of 306 nm. DLE was defined as the ratio of the loaded weight of RSV to the feeding weight of RSV (Equation (1)). DLC was defined as the ratio of the loaded RSV to the total amount of RSV and liposome (Equation (2)).

(1) $$DLE=\frac{weight\;of\;loaded\;drug}{weight\;of\;drug\;in\;feed}\times 100\%$$

(2) $$DLC=\frac{weight\;of\;loaded\;drug}{weight\;of\;drug\;and\;liposome\;in\;feed}\times 100\%$$

### 2.3. Zebrafish Husbandry

For the wound-healing experiments, adult wild-type zebrafish (4–6 months old) were obtained from a reputable aquarium supplier and acclimated in the laboratory before initiating the experiments. The fish were housed in a recirculating tank system and maintained under stable conditions with a 14:10-h light/dark cycle. Water temperature was controlled at 28.5 °C, with a pH of 7.2–7.6 and conductivity ranging from 300 to 1500 µS. Zebrafish were fed twice daily, once with lab-cultured brine shrimp and once with commercial dry food (Taiwan Hung Kuo Industrial Co., Ltd., Taipei, Taiwan). Only healthy zebrafish with normal body morphology were selected for wounding procedures, regardless of sex. All protocols adhered to the guidelines for the care and use of laboratory animals at Chung Yuan Christian University and were approved by the CYCU Animal Ethics Committee (IACUC Approval Number 112010).

### 2.4. Anesthesia and Recovery Procedures for Zebrafish in Wound Healing Assays

To perform the wound-healing assay, zebrafish were anesthetized with a 0.1% MS-222 (Tricaine Methanesulfonate, Sigma Aldrich, St. Louis, MO, USA) solution to reduce movement and discomfort. Immobilization was achieved within 10 to 30 s, allowing precise handling. Once anesthetized, the fish were placed laterally in an agarose mold to stabilize them and expose the dorsal skin for accurate laser ablation. After the procedure, the zebrafish were transferred to an aerated 10-L recovery tank and observed for approximately 30 min until normal swimming behavior resumed, indicating full recovery. This protocol ensured animal ethical treatment while providing consistent, reproducible results.

### 2.5. Generate a Skin Wound Using a Standardized Laser-Based Method

**Figure 1:**
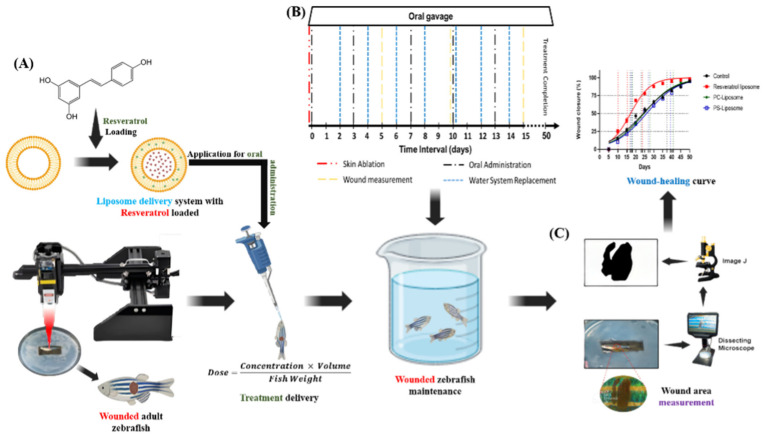
Zebrafish skin injury and wound healing assessment: (**A**) workflow for evaluating liposomal wound healing; (**B**) experimental timeline: red line, skin ablation (day 0); black line, oral dosing (from day 0, twice weekly); blue lines, water replacement (every 2 days); yellow lines, wound measurement points; and (**C**) method for wound size calculation.

In this study, a laser-based skin ablation technique (Figure 1A) was employed in zebrafish based on previous research that has optimized the method using a high-power CO_2_ laser integrated with a laser engraving machine featuring precise XY motorized control [17,18]. Optimized parameters—3 watts of power and approximately 37 s of exposure—produced consistent full-thickness wounds (~400 µm deep, 2 mm in diameter) with minimal variability and negligible impact on zebrafish behavior, ensuring animal welfare and compliance with ethical standards. This approach overcomes the limitations of conventional wound models by providing high reproducibility and precision, in alignment with the 3Rs principles (replace, reduce, refine). This advanced system provides a reliable platform for investigating the therapeutic potential of liposomal RSV in skin wound healing, thereby advancing regenerative medicine research.

### 2.6. Wound Treatment with PC-RSV Liposome and Wound Size Calculation

Adult zebrafish (4–6 months old, mixed gender) were randomly assigned to experimental groups (n = 12 each). Following a previously described protocol, fish were acclimated in a 10 L water system for 5 min and then anesthetized with 0.1% MS-222 for 30 s. A full-thickness laser wound was created on the right flank, anterior to the anal and dorsal fins. After 5 min of recovery, the fish were transferred to experimental tanks, with water changes performed every 2 days (Figure 1B). PC-RSV liposome was administered orally via gavage; anesthesia was confirmed by loss of the righting reflex and absence of a touch response before dosing. The dose for each fish was calculated as:

$$Dose=\frac{Concentration\times Volume}{Fish\;Weight}$$

A fine micropipette was used to deliver PC-RSV liposome into the mouth and pharynx without regurgitation. After dosing, the fish were monitored in a recovery tank until normal swimming behavior resumed, then returned to their home tanks. A reference dose of 1 mg/kg was selected based on previous zebrafish studies demonstrating efficacy and safety across biomedical applications [22]. Treatments were administered weekly in a consumable solution for systemic exposure over 50 days (Figure 1B), while control groups received ddH_2_O on the same schedule to maintain consistency. Additionally, to ensure that observed differences were attributable to treatment effects rather than procedural variation, all experimental conditions were standardized across control and treatment groups (PC, PS, and PC-RSV), including laser ablation, oral administration, anesthesia, housing conditions, feeding, oxygen supply, water replacement, and the experimental timeline.

Wound areas were measured at set intervals, e.g., 5, 10, 15, 20, 25, 30, 35, 40, 45, and 50 days post-laser injury (dpi), to track healing progression. At each time point, zebrafish were anesthetized to minimize stress and movement during imaging. High-resolution wound images were captured using a dissecting microscope (Andonstar, Shenzhen, China) for detailed analysis. Wound areas were quantified using ImageJ (v1.53k) with calibrated scales to ensure measurement consistency (Figure 1C). Percentage wound closure (WC%) was calculated as a standard metric to evaluate healing over time. The laser ablation technique used for wound induction followed a previously validated, reproducible protocol from our earlier work [17].

### 2.7. Data Analysis

Data analysis was conducted using GraphPad Prism version 8.0.2 (GraphPad Inc., La Jolla, CA, USA). An unpaired *t*-test was used to compare the treatment groups with the control group to assess statistical significance. Nonlinear regression was used to model wound closure rates at 25%, 50%, and 75% and to predict progression trends. For multiple group comparisons, two-way ANOVA with Geisser–Greenhouse correction was performed, followed by Dunnett’s multiple comparisons test to identify significant differences between treatment groups and controls [18]. Statistical significance levels were set as follows: * *p* < 0.05, ** *p* < 0.01, *** *p* < 0.001, and **** *p* < 0.0001.

## 3. Results

### 3.1. Physicochemical Properties of Liposomes

**Figure A1:**
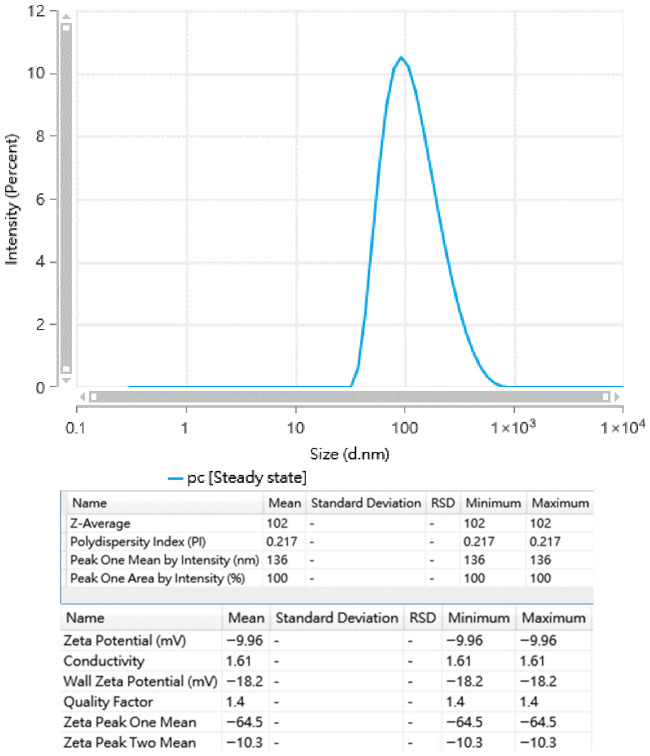
Original particle size histogram and Zeta potential data of PC liposome measured by Zetasizer Ultra (Malvern, UK).

Freshly prepared liposomes were characterized based on average particle size, size distribution (polydispersity index, PI), surface charge (Zeta potential), and loading capacity. The particle size histogram and Zeta potential data for PC, PS, PC-RSV, and Nanosphere^TM^ Size Standards are shown in Figure A1 through A4. These data are summarized in Table 2. For the PC liposome, in which a single lipid component (PC) was used, the average particle size was 102 nm. When PC was mixed with PS, the particle size of the PS liposome increased to 117 nm. Since RSV was encapsulated in the PC-RSV liposome, an average particle size of 158 nm was obtained. During liposome fabrication, an extruder with porous membranes was used to confine the particle size to 200 nm. Therefore, RSV-loaded liposomes produced in this study fell within the desired particle size. In terms of size distribution (the uniformity of particles), PI values for all prepared liposomes ranged from 0.1 to 0.3, indicating a uniform size distribution. When the PI is below 0.05, the nanoparticles are considered monodispersed. In the present study, the fabricated liposomes exhibited a moderate size distribution.

Regarding surface charges, PC, a neutral phospholipid, combined with cholesterol resulted in a negative zeta potential of −9.96 mV for the PC liposome. When negatively charged PS was incorporated, the liposome exhibited a more negative zeta potential (−59.3 mV) compared to the PC liposome. A negative zeta potential enhances electrostatic repulsion, thereby increasing stability and preventing particle aggregation. When RSV was encapsulated in the liposome, the surface charge remained a negative charge, e.g., −40.4 mV

Among the three liposomal formulations, the PC-RSV liposome was loaded with RSV for wound-healing applications. The encapsulation efficiency (DLE) and the RSV payload (DLC) were 32.9% and 5.98%, respectively. DLE represents the percentage of RSV loaded into the PC-RSV liposome relative to the total RSV used during preparation, while DLC indicates the actual amount of RSV loaded in the liposome relative to the liposome formulation.

### 3.2. Image Analysis of Zebrafish Wounds Post-Injury

**Figure 2:**
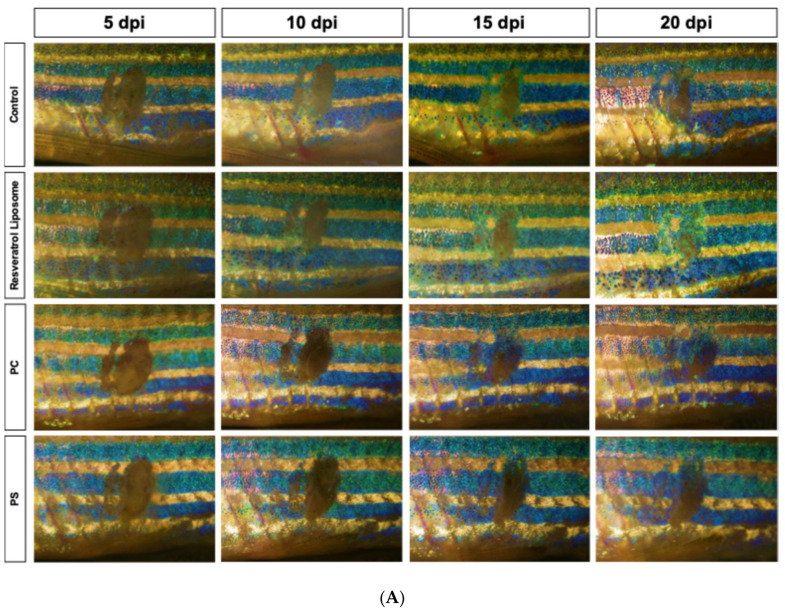

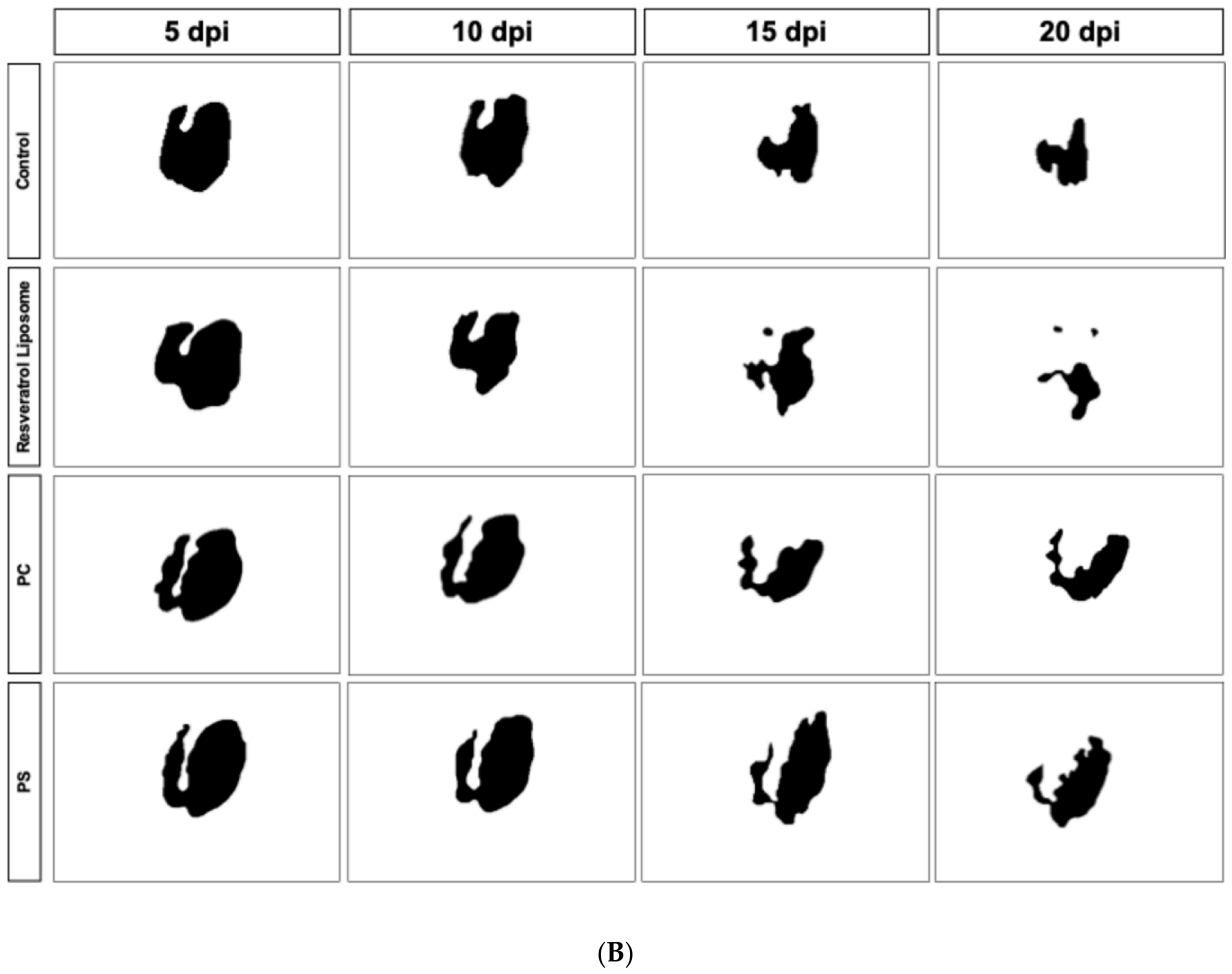
Evaluation of wound healing in zebrafish: (**A**) comparison of RSV-loaded liposome (PC-RSV), phosphatidylserine (PS) liposome, phosphatidylcholine (PC) liposome, and control treatments over 20 days post injury (dpi); and (**B**) corresponding binary segmentation masks illustrate the wound areas, where white represents the wound area and black represents the background tissue.

Figure 2 illustrates the effects of various liposomal formulations on zebrafish skin wound healing over a 20-day post-injury period, which is considered the optimal timeframe for assessing treatment efficacy. Zebrafish treated with the PC-RSV liposome exhibited significantly faster wound closure, with notable reductions in wound size observed by 10 days post-injury (dpi) and near-complete re-epithelialization by 20 dpi.

In contrast, empty liposomes (PC and PS liposomes) showed no significant improvement, indicating that the therapeutic effect was driven by RSV rather than the drug carrier (liposome). Liposomal encapsulation enhances RSV stability and targeted delivery, thereby promoting keratinocyte migration and proliferation, while oral administration provides a minimally invasive approach for evaluating systemic effects, highlighting its potential for managing chronic wounds.

### 3.3. Evaluation of PC-RSV Liposome, PC Liposome, and PS Liposome in Enhancing Wound Healing Efficiency in Zebrafish

**Figure 3:**
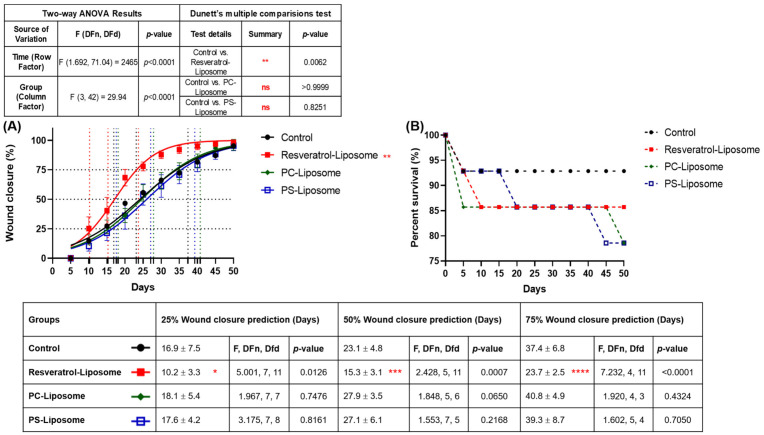
(**A**) Evaluation of resveratrol-loaded liposome, PC (L-α-phosphatidylcholine) liposome, and PS (phosphatidylserine) liposome in promoting skin wound healing in zebrafish following laser ablation; (**B**) Survival rates of each group during the experimental period. Data are presented as mean ± SD. Wound-closure rates were analyzed using two-way ANOVA with Geisser-Greenhouse correction, followed by Dunnett’s test for comparisons with the control group. Wound-closure stages (25%, 50%, and 75%) were estimated by nonlinear regression, and group comparisons were performed using unpaired *t*-tests. Each group included 10–12 zebrafish. Statistical significance: ns: not significant, * *p* < 0.05, ** *p* < 0.01, *** *p* < 0.001, **** *p* < 0.0001.

To establish an effective zebrafish wound-healing model, the therapeutic potential of resveratrol-loaded liposome (PC-RSV) was evaluated alongside blank liposomes (PC and PS) and a control group. Wound closure was monitored at early (25%), mid (50%), and late (75%) stages of healing over 50 days. PC-RSV liposome exhibited the most accelerated healing, achieving 25% closure in 10.2 days compared to 16.9 days in the control group. Similarly, 50% closure occurred in 15.3 days versus 23.1 days in the control group, and 75% closure was reached in 23.7 days, which was significantly faster than the control group’s 37.4 days (Figure 3A).

Blank liposomes (PC and PS) did not differ significantly from the control group, reaching 25–75% closure at similar or slightly delayed time points, confirming effective drug loading and highlighting the role of RSV, rather than the liposomal carrier, in promoting wound repair. Notably, the RSV-loaded formulation consistently outperformed all other groups across all healing stages, emphasizing its ability to enhance skin regeneration throughout the wound-healing process. Survival rate data (Figure 3B) further support these findings. Although the control group exhibited the highest survival rate, wound healing remained slow. In contrast, the PC-RSV liposome balanced both outcomes, achieving markedly faster tissue regeneration with acceptable survival (>85%) throughout the study. These findings highlight the potential application of liposomal RSV as a bioactive wound treatment strategy in zebrafish and potentially broader therapeutic contexts.

## 4. Discussion

### 4.1. Distribution of RSV in Liposomes and Its Association with Liposomal Properties

The chemical structure of the loading drugs plays a crucial role in determining liposome properties. When RSV is encapsulated in liposomes, the core serves as the hydrophilic phase, and the lipid layer constitutes the hydrophobic phase. The distribution of RSV within the liposomes depends on the liposomal formulations, e.g., the type of lipids. Meleleo et al. used electrophysiological measurements to explore interactions between lipids and RSV in a planar lipid model [23]. They reported that the hydroxy group at C3 of cholesterol (CH) was located in the head group region of palmitoyl-oleoyl-phosphatidylcholine (POPC), while the isooctyl group (C17) was positioned within the lipid region of POPC. Notably, RSV was distributed in the lipid region of the planar lipid model and exhibited antioxidant effects primarily through its localization in the hydrophobic lipid layer.

Lipids also influence the rigidity of the liposome membrane. Tsuchiya et al. reported that lower concentrations of CH and higher concentrations of unsaturated PC led to reduced fluidity of the liposome membrane [24], as revealed by measurements of fluorescent polarization. When 1.25–10 μM of RSV were encapsulated in the liposomes, the hydrophobic region of the lipid bilayer was rigidified, indirectly indicating the distribution of RSV in this region. Therefore, when RSV and other flavonoids were supplied as anti-cancer dietary factors in their study, the cellular membranes of cancer cells became rigidified. As a result, oral administration of liposomal RSV may be beneficial in cancer therapy.

Regarding liposome fabrication, Basavarajar et al. reported that higher amounts of CH and PC stabilize the colloidal integrity of liposomes, as evidenced by more negative zeta potentials and smaller particle sizes [25]. In the present study, the zeta potential became more negative from PC liposome to PS liposome. Similary, PC-RSV liposomes exhibited a negative zeta potential compared to the PC liposome because of the addition of PS (Table 2). Notably, RSV can produce a negative surface charge on liposomes compared to PC lipid does during liposome fabrication.

### 4.2. Zebrafish as a Promising Animal Model to Investigate Wound Therapeutics

Adult zebrafish (*Danio rerio*) are a valuable translational model for studying skin wound healing and evaluating therapeutic strategies such as RSV-loaded liposomes. Their genetic and physiological similarity to humans, mature immune and organ systems, and robust regenerative capacity make them suitable for modeling complex pathologies, including chronic inflammation and metabolic disorders [26]. Adult zebrafish provide a robust system for monitoring long-term physiological and behavioral effects [27]. With high-resolution imaging, transparent adult zebrafish enable real-time, non-invasive monitoring of vascular remodeling, immune cell movement, and drug distribution in vivo [17,28], thereby supporting spatiotemporal evaluation of wound healing and treatment efficacy [15]. RSV, a natural polyphenol with antioxidant, anti-inflammatory, and pro-regenerative properties, shows therapeutic potential but is limited by poor solubility, instability, and rapid metabolism [29,30]. Liposomal encapsulation of RSV improves its stability, bioavailability [9], and targeted delivery, and adult zebrafish provide a cost-effective, high-throughput in vivo platform for evaluating these formulations and their systemic effects [31]. Thus, adult zebrafish represent a suitable model for advancing RSV-loaded liposomes and other nanomedicine-based therapies.

### 4.3. Oral Administration of the Therapeutic Agents

Oral administration of RSV faces major pharmacokinetic challenges due to its poor systemic bioavailability, despite high intestinal absorption. Clinically, less than 1% of orally ingested RSV reaches circulation because of extensive first-pass metabolism, limiting its therapeutic value when delivered in free form [9]. To address this, nanoscale carriers have shown translational promise. Previous studies demonstrated that zein nanoparticle formulations significantly improved oral bioavailability in humans [10]. Similarly, encapsulation in lipid-based nanocarriers, such as liposomes, provides an effective strategy to overcome the limitations of RSV. Liposomal formulations enhance aqueous solubility and chemical stability, protect against rapid enzymatic degradation, and promote gastrointestinal absorption [31].

In the zebrafish model, oral gavage has been validated as a precise and reproducible method for systemic drug administration, particularly for biomaterial-based therapies [18]. Building on this rationale, oral liposomal RSV significantly protect degeneration of nerve system in adult zebrafish by enhancing systemic bioavailability and activating regenerative pathways [32]. Liposomes not only achieve higher intestinal stability and absorption [33] but also facilitate cellular uptake via fusogenic interactions, improving neurovascular function for the prevention of age-related cerebromicrovascular pathologies [34]. Given zebrafish’s small size and efficient circulation, systemic delivery ensures rapid biodistribution to injured tissues [35]. Collectively, these findings highlight orally-administered liposomal RSV as a novel, translational therapeutic approach that overcomes the intrinsic pharmacokinetic barriers of RSV and enables systemic regenerative action.

### 4.4. Therapeutic Potential of RSV-Loaded Liposome on Wound Healing

As mentioned in Section 3.1, the negative surface charge (Zeta potential) of the liposome reflected the formulation stability. The PC-RSV liposomes exhibited a negative zeta potential of −40.4 mV, which prevents the particles from aggregating during the dosing period (0–13 days of oral administration, Figure 1). In case of significant degradation during the dosing period, the therapeutic outcomes would likely have mirrored the control groups. However, the results showed that PC-RSV liposomes significantly accelerated healing, reaching 75% wound closure in 23.7 days compared to 37.4 days in the control group. Furthermore, we confirmed that blank liposomes (without RSV) did not improve healing, indicating that the stabilized RSV (PC-RSV liposomes) was the active driver of the observed tissue regeneration.

While zebrafish exhibit rapid scar-free skin regeneration, our study was specifically designed to distinguish treatment-induced acceleration from natural healing. Each treatment group was directly compared with the untreated control using appropriate statistical analyses as mentioned in Section 2.7 (Data Analysis).

Thus, the main experimental variable was the administered formulation (PC, PS, or PC-RSV). The control group received water, the same vehicle used for liposome preparation, thereby controlling for potential gavage and solvent effects. Importantly, blank liposome groups (PC and PS) showed wound-closure kinetics comparable to controls, confirming that neither the vehicle (PC and PS liposomes) nor procedural factors affected regeneration.

In contrast, the PC-RSV group demonstrated significantly accelerated wound closure, reaching 25%, 50%, and 75% closure at 10.2, 15.3, and 23.7 days, respectively, compared to 16.9, 23.1, and 37.4 days in controls (Figure 3). Rather than relying solely on endpoint outcomes, we performed longitudinal quantitative analysis over 50 days and applied nonlinear regression modeling to compare healing kinetics, enabling robust detection of differences in regeneration velocity.

Although untreated zebrafish ultimately achieved wound closure due to their intrinsic regenerative capacity, the substantially shortened healing timeline in the PC-RSV group demonstrates a true treatment-enhanced acceleration beyond baseline healing. Notably, the rapid regenerative capacity of adult zebrafish is also an experimental advantage, as it allows treatment effects on skin wound healing to be observed within a shorter timeframe compared to rodent models.

RSV is well recognized for its multifaceted regenerative actions, including antioxidant and anti-inflammatory effects. In zebrafish larvae, it significantly downregulates injury-induced Mpx and Cox-2 expression, demonstrating early in vivo modulation of inflammation during wound healing [36]. In rat models, oral administration of RSV enhances tensile skin strength, collagen deposition, fibroblast proliferation, and neovascularization, highlighting its systemic therapeutic efficacy [37]. Under negative conditions such as diabetes, RSV promotes angiogenesis and wound closure via the PI3K–AKT–Nrf2 pathway [38], and RSV-loaded hydrogels [39] and scaffolds [40] enhance re-epithelialization, collagen deposition, and anti-inflammatory responses. Pandey et al. reported that enzymatic antioxidants, such as SOD and GSH, in rats were further elevated. Notably, these therapeutic agents were topically applied in rats and mice, whereas the present study employed a different route (oral administration), which is expected to improve patient compliance in human clinical practice.

Building on this background, this study introduces a novel oral liposomal strategy that integrates the systemic bioavailability advantages of liposomes with RSV regenerative bioactivity. Encapsulation improves solubility, stability, and targeted release, enabling efficient biodistribution and wound-site uptake. Mechanistically, RSV reduces oxidative stress via Nrf2 activation [41], modulates inflammation and macrophage activity to drive the proliferative phase [42], and stimulates angiogenesis through the SIRT1–FOXO1–c-Myc pathway [8,43]. These actions complement zebrafish’s inherent regenerative traits, including rapid re-epithelialization and minimal scarring [14,44]. In contrast, blank liposomes such as PC and PS liposomes provide structural or signaling roles but lack sufficient activity to regulate oxidative stress, inflammation, or fibroblast proliferation [45,46]. Consequently, they showed limited wound-healing effects compared to PC-RSV liposomes (Figure 3A), which robustly modulate macrophage recruitment, angiogenesis, and growth factor signaling. Collectively, these findings highlight adult zebrafish as a valuable translational model [15] and establish oral liposomal RSV as a promising systemic nanomedicine-based strategy for treating chronic or non-healing wounds.

### 4.5. The Potential of Clinical Translation of PC-RSV Liposomes

A primary hurdle for the clinical use of free form RSV is its poor systemic bioavailability in mammals. In humans, less than 1% of orally ingested free RSV reaches systemic circulation due to extensive first-pass metabolism and rapid enzymatic degradation [9]. We argue that liposomal encapsulation serves as a translational strategy to address this. Lipid-based carriers protect RSV from degradation in the gastrointestinal tract and promote absorption, thereby achieving higher circulating concentrations than free form RSV [47].

For this study, we utilized a reference dose of 1 mg/kg, administered via oral gavage. This specific dose was selected based on previous zebrafish studies that established its safety and efficacy. The treatment maintained a survival rate of over 85%, indicating that the 1 mg/kg concentration provides a favorable balance between therapeutic speed and systemic safety.

The oral administration is often preferred in human clinical settings over topical applications (like hydrogels or scaffolds) because it can significantly improve patient compliance. Unlike topical treatments that only affect a localized area, the liposomal strategy enables systemic regenerative action, which may be more effective for chronic or complex non-healing wounds where systemic factors (like diabetes) are involved. The biological pathways activated by RSV in this study, such as the SIRT1–FOXO1–c-Myc and PI3K–AKT–Nrf2 pathways are genetically conserved between zebrafish and humans, supporting the relevance of these findings for human regenerative medicine [38].

### 4.6. Limitations and Future Directions

This study demonstrates the therapeutic potential of our RVS-liposome formulation in promoting skin wound healing in adult zebrafish. We applied systemic delivery of RSV via oral route and demonstrated its wound-healing performance using a standardized zebrafish model. However, several limitations should be considered. Biosafety assessment in this study was limited to survival, morphological development, and behavioral observations, and future work should expand these evaluations. Detailed molecular analyses, such as collagen deposition, angiogenesis, inflammatory cell dynamics, and extracellular matrix remodeling, were not comprehensively characterized. Incorporating gene and protein expression profiling of key inflammatory and angiogenic markers would provide deeper mechanistic insight into how liposomal resveratrol modulates tissue repair.

Although the sample size (n = 12 per group) was sufficient to detect statistically significant differences [17,18], larger cohorts could improve robustness and allow stratified analysis of biological variability. Dose–response studies and long-term safety assessments would also help optimize therapeutic regimens. While zebrafish provide valuable insights, differences in skin structure, such as the absence of hair follicles and a multilayered dermis, and variations in immune complexity may limit direct translation to mammals. Therefore, complementary studies in higher vertebrate models are essential for clinical relevance. Future investigations should also include histopathological analyses, long-term evaluations, and mechanistic studies of signaling pathways involved in inflammation and angiogenesis to fully understand therapeutic effects and potential adverse responses.

## 5. Conclusions

This study demonstrates that the oral administration of liposomal resveratrol significantly enhances the skin wound-healing process. By successfully fabricating RSV-loaded liposome with an average particle size of 158 nm and a stable negative surface charge, the typical pharmacokinetic barriers of resveratrol, such as poor bioavailability and chemical instability, were overcome. The scientific findings reveal that PC-RSV liposomes significantly accelerate zebrafish wound closure compared to control and blank liposome groups (PC and PS liposomes). Key healing results were achieved much earlier in the treatment group, with 75% wound closure reached in 23.7 days, compared to 37.4 days for the control group. This rapid regeneration was achieved while maintaining a high survival rate of over 85%, highlighting the safety and efficacy of the liposomal formulation. Furthermore, the study validates the adult zebrafish laser-ablation model as a highly reproducible and translationally relevant platform for the real-time evaluation of nanomedicines. Ultimately, this oral liposomal strategy could leverage the antioxidant and pro-regenerative bioactivity of RSV to provide a potent, systemic approach for managing chronic or non-healing wounds.

## Figures and Tables

**Table 1:** The ingredients of the liposomes. The weight ratios of each liposome are listed in the table. PC: phosphatidylcholine, PS: phosphatidylserine, CH: cholesterol, resveratrol: RSV.

Samples	Weight Percent (%)			
	PC	PS	CH	RSV
PC liposome	8	0	2	0
PS liposome	3	6	1	0
PC-RSV liposome	8	0	2	1

**Table 2:** The particle size, size distribution, and the encapsulation capacity of RSV in liposomes.

Sample	Average Particle Size * (nm)	PI	Zeta Potential (mV)	DLE (%)	DLC (%)
PC liposome	102	0.217	−9.96	-	-
PS liposome	117	0.143	−59.3	-	-
PC-RSV liposome	158	0.207	−40.4	32.90	5.98

* reported as Z-average by DLS instrument (Zetasizer Ultra).

**Figure A2:**
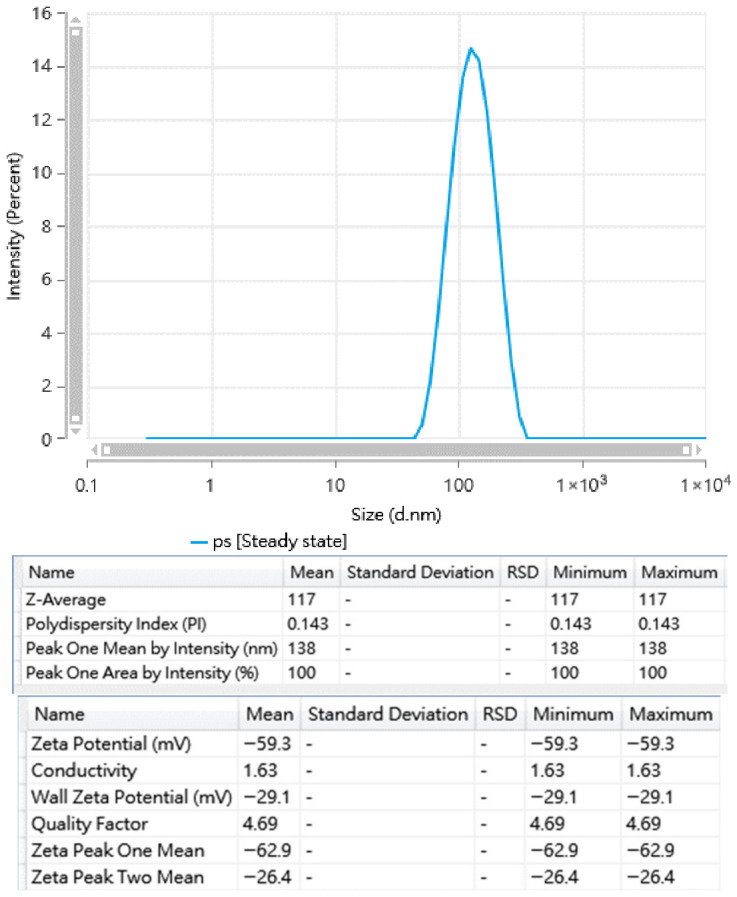
Original particle size histogram and Zeta potential data of PS liposome measured by Zetasizer Ultra (Malvern, UK).

**Figure A3:**
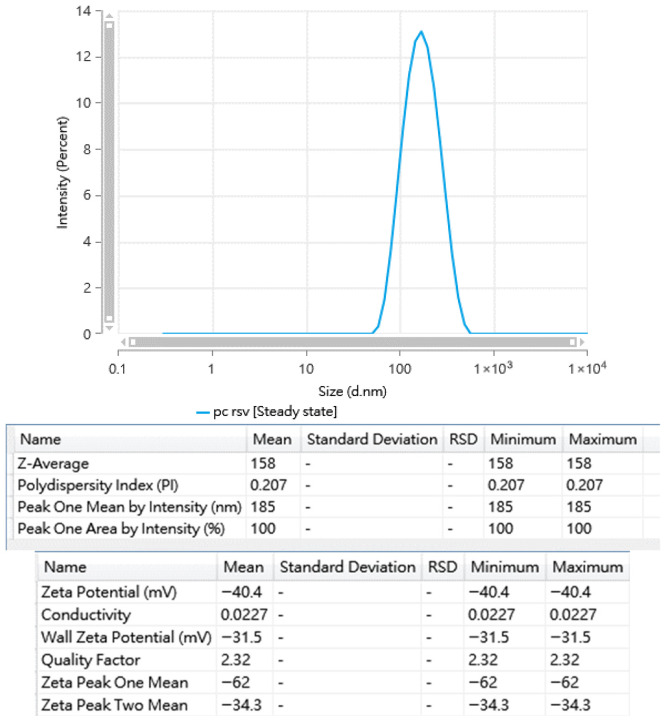
Original particle size histogram and Zeta potential data of PC-RSV liposome measured by Zetasizer Ultra (Malvern, UK).

**Figure A4:**
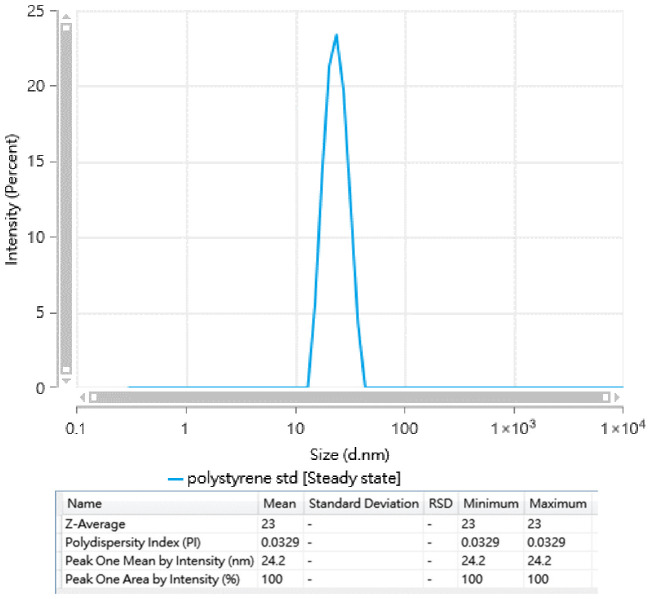
Original particle size histogram of Nanosphere^TM^ Size Standards measured by Zetasizer Ultra (Malvern, UK).

## Data Availability

The original contributions presented in this study are included in the article. Further inquiries can be directed to the corresponding author.

## Abbreviations

The following abbreviations are used in this manuscript:

CH	Cholesterol
DLC	Drug load content
DLE	Drug load efficiency
DLS	Dynamic light scattering
EGF	Epidermal growth factor
HGF	Hepatocyte growth factor
NO	Nitric oxide
PC	Phosphatidylcholine
PI	Polydispersity index
POPC	Palmitoyl-oleoyl-phosphatidylcholine
PS	Phosphatidylserine
RSV	Resveratrol
VEGF	Vascular endothelial growth factor
